# Prevalence of prescribed benzodiazepine long-term use in the French general population according to sociodemographic and clinical factors: findings from the CONSTANCES cohort

**DOI:** 10.1186/s12889-019-6933-8

**Published:** 2019-05-14

**Authors:** Guillaume Airagnes, Cédric Lemogne, Adeline Renuy, Marcel Goldberg, Nicolas Hoertel, Yves Roquelaure, Frédéric Limosin, Marie Zins

**Affiliations:** 1 Department of Psychiatry and Addictology, Hôpitaux Universitaires Paris Ouest, AP-HP, Paris, France; 2 Faculté de Médecine, Université Paris Descartes, Sorbonne Paris Cité, Paris, France; 3 UMS 011, Population-based Epidemiological Cohorts, Inserm, Villejuif, France; 4 UMR 1168, VIMA, Inserm, Villejuif, France; 5 U 894, Centre Psychiatrie et Neurosciences, Inserm, Paris, France; 6 UMR 1085, Ester, Irest Inserm, Université d’Angers, Angers, France; 7 Centre Ambulatoire d’Addictologie, Hôpital Européen Georges Pompidou, 20 rue Leblanc, 75015 Paris, France

**Keywords:** Benzodiazepine, Prevalence, Long-term use, Misuse, General population, Sociodemographic factors, Depression, Alcohol use, French national cohort, Administrative registries

## Abstract

**Background:**

Data are lacking regarding the prevalence of benzodiazepine long-term use in the general population. Our aim was to examine the prevalence of prescribed benzodiazepine long-term use (BLTU) according to sociodemographic and clinical factors in the French general population.

**Methods:**

Data came from 4686 men and 4849 women included in 2015 in the French population-based CONSTANCES cohort. BLTU was examined using drug reimbursement administrative registries from 2009 to 2015. Analyses were weighted to provide results representative of the French general population covered by the general health insurance scheme. Weighted prevalence of BTLU and weighted Odds Ratios (OR) of having BTLU were computed with their 95% Confidence Interval (95% CI) according to age, education level, occupational status, occupational grade, household income, marital status, alcohol use disorder risk and depressive symptoms. All the analyses were stratified for gender.

**Results:**

Weighted prevalence of BLTU were 2.8%(95% CI:2.3–3.4) and 3.8%(95% CI: 3.3–4.5) in men and women, respectively. Compared to men, women had an increased risk of having benzodiazepine long-term use with OR = 1.34(95% CI = 1.02–1.76). Aging, low education, not being at-work, low occupational grade, low income, being alone and depressive state were associated with increased risks of having BTLU.

**Conclusions:**

BLTU is widespread in the French general population, however this issue may particularly concern vulnerable subgroups. These findings may help in raising attention on this public health burden as well as targeting specific at-risk subgroups in preventive intervention.

**Electronic supplementary material:**

The online version of this article (10.1186/s12889-019-6933-8) contains supplementary material, which is available to authorized users.

## Background

Benzodiazepines are the most prescribed drugs worldwide and they are primarily used for their anxiolytic properties [1]. For instance, in the US, alprazolam was the most prescribed of all drugs in 2013 [2]. In France, 20% of the entire population has used a benzodiazepine at least once in 2010 [3]. This consumption represented 124 billion of boxes, and 3.8% of the total drug consumption in 2010 [3]. Although the indication for chronic use only concerns rare conditions such as dystonic syndromes [4, 5], benzodiazepine long-term use (i.e. several weeks of continuous consumption) is a very common phenomenon in number of countries, including those in which benzodiazepines can only be purchased by prescription, such as in France [1, 6–8]. International guidelines, which are based on experts’ consensus, differ regarding the recommended maximum duration of prescription, e.g. 4 weeks in UK and 12 weeks in France, regarding usual indications such as sleep disorders or anxiety [9, 10]. Despite of these guidelines, the median duration of treatment is often much higher, such as 7 months among the French general population in 2010 [3]. The reasons for this long term use are still unclear, although they include at least, from patients and prescribers, an insufficient dissemination and knowledge of the guidelines that are therefore insufficiently implemented, and an underestimation of the harmful consequences of long-term benzodiazepine use by both prescribers and patients [6]. However, long-term exposure to benzodiazepine is associated with substantial side effects, including risk of dependency and related symptoms (e.g. craving, withdrawal symptoms) and other potentially life-threatening side effects such as increased risks of falls, car crash and respiratory failure [1, 11]. In addition, it is noteworthy that several withdrawal symptoms can be perceived as the resurgence of the symptoms that indicated the initial prescription of benzodiazepine (e.g. sleep disorders or anxiety), leading to the resumption of benzodiazepine use [12]. Long-term benzodiazepine use has also been associated with psychological side effects (e.g. cognitive impairments, depressive mood, increased impulsivity and suicidal behaviors, sleep disorders) and with specific diseases (e.g. cancers, Alzheimer disease) [1, 13]. To prevent these detrimental effects, it is therefore crucial to describe the burden of benzodiazepine long-term use at a population level in order to target at-risk subgroups and to adjust screening and preventive strategies according to the assessment of their efficiency, based on changes in prevalence.

However, data are lacking in the literature regarding the prevalence of benzodiazepine long-term use in the general population [3]. When available, these data are usually self-reported [14, 15]. Indeed, screening may not be easily performed in primary care [16]. Although self-report might be reliable in some areas (e.g. education), this reliability might be questioned when it comes to benzodiazepines long-term use. A lack of knowledge about the type of medication used, social desirability or fear of stigma associated with mental disorders, fear of disclosing having several prescribers or that such disclosure may lead to reduced access to benzodiazepines are examples of biases that may specifically contribute to benzodiazepines long-term use misreporting, even during targeted screening [17]. Moreover, remembering having taken benzodiazepine for more or less than 12 weeks in the previous years could be particularly prone to recall bias, especially for drugs with many different trade and molecule names. Only few studies were based on objective reporting (e.g. administrative registries) and their descriptive analyses were mainly presented in Defined Daily Doses (DDD) per 1000 participants per day [3]. This statistical tool is useful to compare global consumptions between two samples, but cannot inform on the prevalence of benzodiazepine long-term use because this indicator vary with both intensity of consumption and duration of use per patient [18]. In addition, dataset with objective reporting often lack sociodemographic and clinical variables, thus providing no descriptive analyses according to at-risk subgroups [19]. However, prior studies have found that benzodiazepine consumers may be more prone to be women, of low occupational grade, over middle-age, and with chronic diseases [3, 20, 21]. However these data have been less explored in the subgroup having the highest risk of side effects, i.e. long-term users [22]. Beyond age, gender and occupational grade, other sociodemographic factors such as education, occupational status, income and marital status could also be associated with benzodiazepine long-term use [9, 22]. These associations would be in line with those found for other types of substances, such as alcohol and tobacco use [23]. As regards clinical factors, benzodiazepine long-term use might be more common among individuals with depression [24, 25] or alcohol use disorder [26]. Consequently, an estimate of the prevalence of benzodiazepine long-term use in the general population while stratifying for sociodemographic and clinical factors would be particularly useful to target at-risk populations for both screening and preventive strategies.

The CONSTANCES cohort includes a large randomized sample of the French population, from various sociodemographic status [27]. Its linkage with national databases of reimbursed drugs offers the opportunity to collect the data regarding all prescribed treatments as soon as the patient has purchased them from the pharmacy [19]. Weighted analyses could be performed in order to provide results representative of the French general population covered by the general health insurance scheme [28]. Therefore we took advantage of the CONSTANCES cohort to examine the prevalence of benzodiazepine long-term use in the French general population according to sociodemographic and clinical factors.

## Methods

### Participants

The CONSTANCES cohort is a national population-based cohort of randomly recruited participants, including volunteers aged 18–69 years at baseline in 22 selected health screening centers from the principal regions of France [27]. To be recruited, participants must be covered by the general health insurance scheme restricted to salaried workers, professionally active or retired and their family (more than 90% of the French population), thus excluding agricultural and self-employed workers which are affiliated to other health insurance funds. The inclusion visit comprises a set of self-report questionnaires including social and demographic characteristics. The CONSTANCES cohort has obtained the authorization of the National Data Protection Authority (Commission Nationale de l’Informatique et des Libertés, no. 910486) and was approved by the Institutional Review Board of the National Institute for Medical Research – INSERM (no. 01–011). Written informed consent was received from all of the subjects in the CONSTANCES cohort. In the present study, we selected participants included in 2015 in the CONSTANCES cohort. Thus, a total of 9535 participants (4686 men and 4849 women) have been included in the statistical analyses.

### Procedure of identification of a benzodiazepine long-term use

The CONSTANCES cohort benefits from its systematic linkage to the SNIIRAM (“Système national d’information inter-régimes de l’Assurance maladie”) database [27]. This national administrative database contains detailed individual medical data for almost the whole French population, and in particular reimbursement data including prescribed drugs [29]. At the time of the study, reimbursement data for CONSTANCES participants were available from January 2009 to December 2015. Filled prescriptions for all benzodiazepines having a marketing authorization in France were extracted from the database, i.e. prescriptions that patients have been purchased in pharmacies. These benzodiazepines are the following: clonazepam, chlordiazepoxide, oxazepam, potassium clorazepate, lorazepam, bromazepam, clobazam, prazepam, alprazolam, nordazepam, ethyl loflazepate and clotiazepam. Then, we used automated algorithms to search for sequences of delivered prescriptions indicating a continuous period of prescription longer than the maximum duration authorized in France (i.e. 12 weeks) [10, 30]. This long-term exposure, leading to side effects such as dependency, is inappropriate regarding the French good practice recommendations and was used to define benzodiazepine long-term use [10]. In France the packaging of drug boxes corresponds to a treatment period of approximately one month, and the pharmacist is not allowed to deliver more than one month of treatment. For instance, even if the patient has a three-month prescription, the treatment must be delivered every month. Based on this assumption that a prescription usually covers one month, we aimed to determine sequences of delivered prescriptions that were most likely corresponding to a continuous use of more than 12 weeks. First, after an initial delivery followed by two refills in the next 12 weeks, the total duration of use was considered of 12 weeks. Second, since we were interested about subjects whose treatment exceeded this duration, we searched for another refill immediately after these 12 first weeks (i.e. at weeks 13–14) to identify a long-term use. In addition, since those having enough treatment at weeks 13–14 may have purchased their third refill later, we also considered as having a long-term use those with a third refill at weeks 15–16 when the second refill took place during weeks 11–12. Therefore, we considered that the sequences with the following criteria were likely to signal a long-term use: 1) At least two refills in the 12 weeks following the first prescription and 2) at least one refill in week 13–14 or at least one refill in week 15–16 if the last refill observed during the first 12 weeks occurs on week 11–12. Based on this classification, we computed a binary outcome for long-term use (i.e. presence versus absence) [31]. Since administrative records are exhaustive, we had no missing data for the outcome.

### Covariables

From the baseline questionnaires, we used the following sociodemographic variables: age (categorized in three modalities as follows: ≤35; from > 35 to ≤50 and > 50); gender; occupational status (employed or in training, including sick leave, leave without pay or availability, maternity, paternity, adoption, parental leave/job seeking/retired or withdrawn from business/other situations by aggregating the three following categories to prevent from too small number of events: does not work for health reasons (disability, chronic illness, …) (*n* = 81), without professional activity (*n* = 18), other situations (*n* = 16)); occupational grade (never worked/blue-collar worker and craftsman/clerk (e.g. clerical or commercial employee, childcare worker, service agent)/intermediate worker (e.g. school teacher, nurse, technician, foreman, supervising officer)/executive (e.g. engineer, doctor)); household income in euros per month (< 2100/from 2100 to 2800/from 2800 to 4200/> 4200); marital status (single/married or living as a couple/separated, divorced or widowed); education level based on the 2011 International Standard Classification of Education (ISCED) with a categorical variable corresponding to the highest obtained degree [32]. In the present study, we aggregated some categories to prevent from too small sample size, as follows: 1) levels 0 and 1 (early childhood education and primary education) and level 2 (lower secondary education); 2) levels 3 and 4 (upper secondary education and post-secondary non-tertiary education); 3) levels 5 and 6 (short-cycle tertiary education and Bachelor’s or equivalent level), and 4) levels 7 and 8 (Master’s or equivalent level and Doctoral or equivalent level). Depressive symptoms were collected as a continuous variable with the Center of Epidemiologic Studies Depressive state scale (CESD). Since a global score ≥ 19 may signal clinically meaningful depressive state, we used this cut-off to provide a proxy of depressive state [33]. Alcohol use risk categories were defined from the total score at the Alcohol Use Disorders Identification Test (AUDIT) as follows: 1) Mild (0–7); 2) Dangerous (8–15); 3) Problematic (16–19) and 4) Dependence (20–40) [34]. In the present study, we merged the three last categories in order to prevent from too small number of events (*n* = 83, *n* = 18 and *n* = 21, respectively), thus providing a binary variable regarding at-risk alcohol use.

### Statistical analyses

Weighted analyses were performed in order to provide results representative of the French general population covered by the general health insurance scheme. A weighting coefficient has been computed for each subject by the CONSTANCES team [28]. This coefficient took into account both the survey weight and the non-participation correction factor based on the follow-up of a control cohort of non-participants [28]. Firstly, weighted prevalence (in percentages) of benzodiazepine long-term use were computed with their 95% confidence interval (95% CI) in the entire sample of men and women, and then according to each covariable. Secondly, weighted odds ratios of having benzodiazepine long-term use according to each covariable were computed with their 95% confidence interval (95% CI) using two-sided binomial logistic regressions for complex samples. All the analyses were stratified for gender. Included subjects had complete data for benzodiazepine long-term use, age and gender. We had missing data for the other variables (from 1.8 to 17.1%). Assuming a missing at random mechanism, multiple imputation was preferred to complete-case analysis to limit the risk of selection bias [35]. Statistical significance was determined using a two-sided alpha a priori set at 0.05 and analyses were performed with IBM Corp. Released 2013. IBM SPSS Statistics for Windows, Version 22.0. Armonk, NY: IBM Corp.

## Results

Prevalence of benzodiazepine long-term use in the French general population covered by the general health insurance scheme were 2.8%(95% CI = 2.3–3.4) and 3.8%(95% CI = 3.3–4.5) in men and women, respectively. The distributions of the prevalence of benzodiazepine long-term use according to sociodemographic and clinical variables are displayed in Table 1. Detailed data are presented in Additional file 1: Table S1 and Additional file 2: Table S2 for men and women, respectively.

**Table 1:** Prevalence of benzdiazepine long-term use of the French general population in 2015

	MEN				WOMEN			
	N^a^	%^b^	95% CI^c^		N^a^	%^b^	95% CI^c^	
			min	max			min	max
Age								
≥18 and ≤ 35	23	**1.7**	1.0	2.9	32	**2.7**	1.6	4.6
> 35 and ≤ 50	68	**4.9**	3.3	7.3	96	**6.6**	5.1	8.4
> 50	141	**9.3**	7.2	11.9	194	**12.2**	10.0	14.9
Education level^d^								
0–2	58	**14.1**	9.7	19.9	79	**15.1**	11.4	19.8
3–4	111	**6.7**	5.0	8.9	138	**9.1**	7.1	11.5
5–6	41	**3.6**	2.1	6.1	84	**5.1**	3.7	7.0
7–8	22	**1.5**	0.8	2.6	21	**3.4**	1.7	6.8
Occupational status								
Employed or in training	93	**2.4**	1.8	3.2	169	**4.7**	3.8	5.9
Job seeking	21	**4.6**	2.6	8.1	23	**6.7**	3.6	12.3
Retired	58	**8.5**	5.8	12.3	75	**12.3**	8.8	16.9
Other situations	60	**30.6**	21.6	41.4	55	**22.3**	16.2	29.8
Occupational grade								
Never worked	17	**37.7**	21.3	57.5	10	**14.4**	6.8	28.0
Blue-collar worker and craftsman	91	**7.8**	5.8	10.4	34	**8.0**	5.3	12.0
Clerk	51	**5.2**	3.3	8.1	172	**9.0**	7.2	11.1
Intermediate worker	31	**4.3**	2.2	8.1	74	**6.5**	4.7	9.1
Executive	42	**2.6**	1.7	4.0	32	**4.4**	2.6	7.3
Household income (in euros)								
< 2100	122	**11.1**	8.5	14.4	167	**11.5**	9.2	14.3
> 2100 and ≤ 2800	40	**4.6**	2.7	7.8	60	**6.8**	5.0	9.3
> 2800 and ≤ 4200	42	**2.6**	1.7	4.1	66	**5.0**	3.6	6.9
> 4200	28	**2.3**	1.4	3.9	29	**4.2**	2.3	7.4
Marital status								
Single	58	**9.4**	6.4	13.5	52	**6.3**	4.1	9.6
Married or living as a couple	119	**3.7**	2.7	5.0	163	**5.7**	4.6	7.1
Separated, divorced or widowed	55	**11.7**	8.1	16.5	107	**15.8**	12.2	20.2
Alcohol use disorder risk^d^								
Mild	150	**5.1**	4.0	6.5	282	**7.4**	6.2	8.7
At-risk	82	**7.3**	5.1	10.3	40	**8.5**	5.6	12.7
Depressive state^e^								
No	131	**3.4**	2.6	4.4	146	**4.8**	3.7	6.1
Yes	101	**15.0**	11.2	19.7	176	**14.0**	11.4	17.0

^a^N: Unweighted headcount; ^b^Weighted prevalence, presented in bold for better readability; ^c^Confidence Interval at 95% of the weighted prevalence; ^d^Based on the 2011 International Standard Classification of Education; ^d^At-risk alcohol use disorder was defined as a total score > 7 at the Alcohol Use Disorder Identification Test; ^e^Depressive state was defined as a total score > 18 at the Center for Epidemiological Studies Depression Scale. Results were computed from weighted analyses of 4686 men and 4849 women included in 2015 in the CONSTANCES cohort

Compared to men, women had an increased risk of benzodiazepine long term use with OR = 1.34(95% CI = 1.02–1.76). In both men and women, the following conditions were associated with increased risks of benzodiazepine long-term use: age over 35 compared to less; not being in couple; low education; low income; not being in employment; depressive state (all *p* < 0.05; Table 2). We did not found any significant association between alcohol use disorder risk and benzodiazepine long-term use. All the results were weighted to ensure the representativeness of the French general population covered by the general health insurance scheme.

**Table 2:** Odds ratios of benzodiazepine long-term use risk in the French general population in 2015

	Men			Women		
	OR	CI95%		OR	CI95%	
Age						
≥18 and ≤ 35	1	–	–	1	–	–
> 35 and ≤ 50	**2.99**	**1.51**	**5.92**	**2.54**	**1.38**	**4.69**
> 50	**5.87**	**3.20**	**10.75**	**5.05**	**2.78**	**9.17**
Education level^1^						
0–2	**10.89**	**5.39**	**22.01**	**5.04**	**2.29**	**11.09**
3–4	**4.73**	**2.48**	**9.04**	**2.81**	**1.31**	**6.05**
5–6	**2.48**	**1.13**	**5.47**	1.53	0.69	3.37
7–8	1	–	–	1	–	–
Occupational status						
Employed or in training	1	–	–	1	–	–
Job seeking	**2.02**	**1.05**	**3.91**	1.45	0.72	2.93
Retired	**3.85**	**2.31**	**6.42**	**2.81**	**1.82**	**4.33**
Other situations	**18.28**	**10.45**	**31.99**	**5.75**	**3.65**	**9.05**
Occupational grade						
Never worked	**22.5**	**8.9**	**56.4**	**3.66**	**1.34**	**9.98**
Blue-collar worker and craftsman	**3.14**	**1.82**	**5.43**	1.90	0.93	3.85
Clerk	**2.03**	**1.06**	**3.89**	**2.16**	**1.19**	**3.93**
Intermediate worker	1.65	0.73	3.75	1.53	0.80	2.94
Executive	1	–	–	1	–	–
Household income (in euros)						
< 2100	**5.27**	**2.88**	**9.62**	**2.98**	**1.54**	**5.76**
> 2100 and ≤ 2800	2.03	0.84	4.39	1.69	0.84	3.38
> 2800 and ≤ 4200	1.14	0.57	2.29	1.22	0.61	2.44
> 4200	1	–	–	1	–	–
Marital status						
Married or living as a couple	1	–	–	1	–	–
Single	**2.67**	**1.59**	**4.50**	1.12	0.68	1.85
Separated, divorced or widowed	**3.43**	**2.05**	**5.71**	**3.11**	**2.13**	**4.55**
Alcohol use disorder risk^2^						
Mild	1	–	–	1	–	–
At-risk	1.45	0.92	2.31	1.17	0.72	1.90
Depressive state^3^						
No	1	–	–	1	–	–
Yes	**5.03**	**3.26**	**7.77**	**3.25**	**2.30**	**4.58**

*OR* Odd Ratio, *CI95%* Confidence Interval at 95%; ^1^ Based on the 2011 International Standard Classification of Education; ^2^ At-risk alcohol use disorder was defined as a total score > 7 at the Alcohol Use Disorder Identification Test; ^3^ Depressive state was defined as a total score > 18 at the Center for Epidemiological Studies Depression Scale. Benzodiazepine long-term use was defined as at least one episode of long-term use from 2009 to 2015. Results were computed from weighted analyses of 4686 men and 4849 women included in 2015 in the CONSTANCES cohort. Significant results at *p* < 0.05 are presented in bold

## Discussion

To our knowledge, this is the first study that examined prevalence of benzodiazepine long-term use in a national representative population-based cohort while examining men and women separately. Moreover, sample size allowed us to perform stratified analyses for sociodemographic and clinical variables. Finally, benzodiazepine long-term delivery was identified thanks to administrative registries, providing an objective reporting with no missing data.

However, this study has several limitations. First, we could not ascertain that delivered medications were actually used and this method of assessment did not include over-the-counter consumptions. However, going to the pharmacy to get benzodiazepines at least four times without actual intake may be unlikely. Moreover, benzodiazepines cannot be obtained without prescription in France; thus over-the-counter consumption is more restricted than in other countries in which these drugs can be purchased without prescription. Addressing these limitations might require compliance control techniques used in clinical research (e.g. manual or electronic pill counts, repeated blood or urine dosages), that are not well adapted to epidemiology given the very high cost of monitoring thousands of subjects in this way [36]. Second, since weighting coefficients were available only for participants from the general health insurance scheme, the representativeness of our results is restricted to this group. However, this scheme concerns the largest number of individuals in France, i.e. more than 90% [37].

Our findings regarding differences of prevalence according to sociodemographic and clinical factors are in line with results from other cohort studies that found associations between benzodiazepine inappropriate use and gender, age, education, occupational status, occupational grade, income, marital status and depressive symptoms [15, 22, 25, 38]. However, from a public health perspective, we provided weighted prevalence and weighted odds ratios, informing not only on significant associations but also on the number of concerned subjects in a national representative sample. Regarding gender differences, women were found to be more prone to use benzodiazepines in several countries [20, 39]. Several hypotheses in order to explain these differences have been proposed, such as more frequent mood and anxiety disorders among women, combined with a more frequent use of the health care system [21]. In addition, although men often use alcohol to cope with stress or negative emotions, women may be more likely to use medication to deal with these situations [21]. Since our results were based on weighted analyses, interactions between gender and the covariates would have been difficult to interpret and were thus not searched in the present study that aims to provide nationally representative estimates. However, further studies based on crude data may explore the potential gender differences in the associations between benzodiazepine long-term use and sociodemographic and clinical factors.

The present study shows that benzodiazepine long-term use concerns a huge number of subjects in the French general population. This issue does not seem to be specific to France, with warnings in several other countries such as UK, Norway, Sweden, Germany, Netherlands, Denmark, USA and Australia [4, 6, 12, 14, 25]. Several hypotheses might explain this high prevalence of benzodiazepine long-term use. Regarding prescribers, several reasons may explain why benzodiazepines may be prescribed above the maximum recommended duration. First, patients with benzodiazepine dependence may seek prescription from several prescribers. Second, prescribers often feel helpless when a patient mentions emotional complaints (e.g. stress, anxiety, sleep disorders). The prescription of benzodiazepines appears to be a simple and effective therapeutic response, even when non-drug treatments would be preferable and a more thorough psychiatric evaluation would be necessary [12]. Regarding patients, several reasons may explain their difficulty in benzodiazepine weaning. First, benzodiazepines are particularly prone to induce dependence [4]. They provide a feeling of physical and psychological well-being that is quickly perceived, which can lead to their compulsive use. There is also a phenomenon of tolerance, so that the decrease in effects can lead to an increase in doses and therefore an increased risk of dependence [40]. After several weeks of treatment, the occurrence of withdrawal symptoms at cessation can lead to a resumption of treatment, especially if these symptoms are not interpreted as withdrawal but as the reappearance of symptoms for which the treatment was initially prescribed [12]. Second, even without becoming dependent, some people may use benzodiazepines to help them to cope with stress or for performance-enhancing [12, 31, 41]. Third, some chronic mental disorders may be mistreated with benzodiazepines [1]. For example, previous findings have shown that many patients with major depressive syndrome receive benzodiazepines instead of an antidepressant [42]. Future research using qualitative methodologies would be useful to better understand the motivations of both patients and prescribers to use benzodiazepines chronically.

To prevent from detrimental consequences of chronic use, several guidelines have been published to help practitioners, including how to perform screening and managing withdrawal [43–45]. However, only slight prevalence decreases have been observed these years (e.g. in France, between 2012 and 2015, there was a decrease of 3.8% in the prevalence of at least one consumption in the year) [3, 46]. Our findings may be useful to build public health information and prevention campaigns for the general public by targeting specific at-risk subgroups according to sociodemographic factors. Further studies should focus on specific groups not included in the general social security scheme, such as farmers or self-employed workers. Since several side effects of benzodiazepine long-term use might differ according to dosages (e.g. cognitive impairments, sedation), future studies should take into account not only duration of consumption but also dosages [47].

## Conclusions

Prevalence of prescribed benzodiazepine long-term use in the French general population were 2.8%(95% CI = 2.3–3.4) and 3.8%(95% CI = 3.3–4.5) in men and women, respectively. Aging, poor sociodemographic conditions and depressive state were significantly associated with increased risks of benzodiazepine long-term use. These findings, based on weighted analyses from participants included in 2015 in the national population-based CONSTANCES cohort, informed not only on significant associations but also on the number of concerned subjects. Thus, benzodiazepine long-term use is widespread in the French general population, however this issue may particularly concerns vulnerable subgroups. These findings may help in raising attention on this public health burden as well as targeting specific at-risk subgroups in preventive intervention.

## Additional files

Additional file 1: **Table S1.** Prevalence of benzodiazepine long-term use in men of the French general population in 2015. (DOCX 26 kb)

Additional file 2: **Table S2.** Prevalence of benzodiazepine long-term use in women of the French general population in 2015. (DOCX 26 kb)

## Abbreviations

AUDIT: Alcohol use disorders identification test
BLTU: Benzodiazepine long-term use
CESD: Center of epidemiologic studies depressive state scale
CI: Confidence interval
INSERM: National institute for medical research
ISCED: International standard classification of education
OR: Odd ratio
SNIIRAM: “Système national d'’information inter-régimes de l'’Assurance maladie” database
