# Supplementary material for: Prevalence of prescribed benzodiazepine long-term use in the French general population according to sociodemographic and clinical factors: findings from the CONSTANCES cohort
Source: BMC Public Health. 2019 May 14;19:566. doi: 10.1186/s12889-019-6933-8 (PMC6518636; doi:10.1186/s12889-019-6933-8)
Supplement: Supplementary file 1 — Table S1. Prevalence of benzodiazepine long-term use in men of the French general population in 2015. (DOCX 26 kb) [file 12889_2019_6933_MOESM1_ESM.docx]

**Supplemental Table 1. Prevalence of benzodiazepine long-term use in men of the French general population in 2015.**

| **Benzodiazepine long-term use** | **No** | | | | **Yes** | | | |
| --- | --- | --- | --- | --- | --- | --- | --- | --- |
|  | **N^1^** | **%^2^** | **95%CI^3^** | | **N^1^** | **%^2^** | **95%CI^3^** | |
|  |  |  | **min** | **max** |  |  | **min** | **max** |
|  |  |  |  |  |  |  |  |  |
| **Age** |  |  |  |  |  |  |  |  |
| ≥18 and ≤35 | 1261 | **98.3** | 97.1 | 99.0 | 23 | **1.7** | 1.0 | 2.9 |
| >35 and ≤50 | 1534 | **95.1** | 92.7 | 96.7 | 68 | **4.9** | 3.3 | 7.3 |
| >50 | 1659 | **90.7** | 88.1 | 92.8 | 141 | **9.3** | 7.2 | 11.9 |
|  |  |  |  |  |  |  |  |  |
| **Education level^4^** |  |  |  |  |  |  |  |  |
| 0-2 | 450 | **85.9** | 80.1 | 90.3 | 58 | **14.1** | 9.7 | 19.9 |
| 3-4 | 1742 | **93.3** | 91.1 | 95.0 | 111 | **6.7** | 5.0 | 8.9 |
| 5-6 | 1174 | **96.4** | 93.9 | 97.9 | 41 | **3.6** | 2.1 | 6.1 |
| 7-8 | 1088 | **98.5** | 97.4 | 99.2 | 22 | **1.5** | 0.8 | 2.6 |
|  |  |  |  |  |  |  |  |  |
| **Occupational status** |  |  |  |  |  |  |  |  |
| Employed or in training | 3240 | **97.6** | 96.8 | 98.2 | 93 | **2.4** | 1.8 | 3.2 |
| Job seeking | 371 | **95.4** | 91.9 | 97.4 | 21 | **4.6** | 2.6 | 8.1 |
| Retired | 683 | **91.5** | 87.7 | 94.2 | 58 | **8.5** | 5.8 | 12.3 |
| Other situations | 160 | **69.4** | 58.6 | 78.4 | 60 | **30.6** | 21.6 | 41.4 |
|  |  |  |  |  |  |  |  |  |
| **Occupational grade** |  |  |  |  |  |  |  |  |
| Never worked | 46 | **62.3** | 42.5 | 78.7 | 17 | **37.7** | 21.3 | 57.5 |
| Blue-collar worker and craftsman | 1212 | **92.2** | 89.6 | 94.2 | 91 | **7.8** | 5.8 | 10.4 |
| Clerk | 813 | **94.8** | 91.9 | 96.7 | 51 | **5.2** | 3.3 | 8.1 |
| Intermediate worker | 888 | **95.7** | 91.9 | 97.8 | 31 | **4.3** | 2.2 | 8.1 |
| Executive | 1495 | **97.4** | 96.0 | 98.3 | 42 | **2.6** | 1.7 | 4.0 |
|  |  |  |  |  |  |  |  |  |
| **Household income (in euros)** |  |  |  |  |  |  |  |  |
| <2100 | 1223 | **88.9** | 85.6 | 91.5 | 122 | **11.1** | 8.5 | 14.4 |
| >2100 and ≤2800 | 761 | **95.4** | 92.2 | 97.3 | 40 | **4.6** | 2.7 | 7.8 |
| >2800 and ≤4200 | 1343 | **97.4** | 95.9 | 98.3 | 42 | **2.6** | 1.7 | 4.1 |
| >4200 | 1127 | **97.7** | 96.1 | 98.6 | 28 | **2.3** | 1.4 | 3.9 |
|  |  |  |  |  |  |  |  |  |
| **Marital status** |  |  |  |  |  |  |  |  |
| Single | 861 | **90.6** | 86.5 | 93.6 | 58 | **9.4** | 6.4 | 13.5 |
| Married or living as a couple | 3165 | **96.3** | 95.0 | 97.3 | 119 | **3.7** | 2.7 | 5.0 |
| Separated, divorced or widowed | 428 | **88.3** | 83.5 | 91.9 | 55 | **11.7** | 8.1 | 16.5 |
|  |  |  |  |  |  |  |  |  |
| **Alcohol use disorder risk^5^** |  |  |  |  |  |  |  |  |
| Mild | 3285 | **94.9** | 93.5 | 96.0 | 150 | **5.1** | 4.0 | 6.5 |
| At-risk | 1169 | **92.7** | 89.7 | 94.9 | 82 | **7.3** | 5.1 | 10.3 |
|  |  |  |  |  |  |  |  |  |
| **Depressive state^6^** |  |  |  |  |  |  |  |  |
| No | 3703 | **96.6** | 95.6 | 97.4 | 131 | **3.4** | 2.6 | 4.4 |
| Yes | 751 | **85.0** | 80.3 | 88.8 | 101 | **15.0** | 11.2 | 19.7 |
| ^1^N: Unweighted headcount; ^2^Weighted prevalence; ^3^Confidence Interval at 95% of the weighted prevalence; ^4^Based on the 2011 International Standard Classification of Education; ^5^At-risk alcohol use disorder was defined as a total score >7 at the Alcohol Use Disorder Identification Test; ^6^Depressive state was defined as a total score >18 at the Center for Epidemiological Studies Depression Scale. Results were computed from weighted analyses of 4686 men included in 2015 in the CONSTANCES cohort. | | | | | | | | |
