# Supplementary material for: Prevalence of prescribed benzodiazepine long-term use in the French general population according to sociodemographic and clinical factors: findings from the CONSTANCES cohort
Source: BMC Public Health. 2019 May 14;19:566. doi: 10.1186/s12889-019-6933-8 (PMC6518636; doi:10.1186/s12889-019-6933-8)
Supplement: Supplementary file 2 — Table S2. Prevalence of benzodiazepine long-term use in women of the French general population in 2015. (DOCX 26 kb) [file 12889_2019_6933_MOESM2_ESM.docx]

**Supplemental Table 2. Prevalence of benzodiazepine long-term use in women of the French general population in 2015.**

| **Benzodiazepine long-term use** | **No** | | | | **Yes** | | | |
| --- | --- | --- | --- | --- | --- | --- | --- | --- |
|  | **N^1^** | **%^2^** | **95%CI^3^** | | **N^1^** | **%^2^** | **95%CI^3^** | |
|  |  |  | **min** | **max** |  |  | **min** | **max** |
|  |  |  |  |  |  |  |  |  |
| **Age** |  |  |  |  |  |  |  |  |
| ≥18 and ≤35 | 1386 | **97.3** | 95.4 | 98.4 | 32 | **2.7** | 1.6 | 4.6 |
| >35 and ≤50 | 1547 | **93.4** | 91.6 | 94.9 | 96 | **6.6** | 5.1 | 8.4 |
| >50 | 1594 | **87.8** | 85.1 | 90.0 | 194 | **12.2** | 10.0 | 14.9 |
|  |  |  |  |  |  |  |  |  |
| **Education level^4^** |  |  |  |  |  |  |  |  |
| 0-2 | 437 | **84.9** | 80.2 | 88.6 | 79 | **15.1** | 11.4 | 19.8 |
| 3-4 | 1607 | **90.9** | 88.5 | 92.9 | 138 | **9.1** | 7.1 | 11.5 |
| 5-6 | 1597 | **94.9** | 93.0 | 96.3 | 84 | **5.1** | 3.7 | 7.0 |
| 7-8 | 886 | **96.6** | 93.2 | 98.3 | 21 | **3.4** | 1.7 | 6.8 |
|  |  |  |  |  |  |  |  |  |
| **Occupational status** |  |  |  |  |  |  |  |  |
| Employed or in training | 3321 | **95.3** | 94.1 | 96.2 | 169 | **4.7** | 3.8 | 5.9 |
| Job seeking | 385 | **93.3** | 87.7 | 96.4 | 23 | **6.7** | 3.6 | 12.3 |
| Retired | 593 | **87.7** | 83.1 | 91.2 | 75 | **12.3** | 8.8 | 16.9 |
| Other situations | 228 | **77.7** | 70.2 | 83.8 | 55 | **22.3** | 16.2 | 29.8 |
|  |  |  |  |  |  |  |  |  |
| **Occupational grade** |  |  |  |  |  |  |  |  |
| Never worked | 73 | **85.6** | 72.0 | 93.2 | 10 | **14.4** | 6.8 | 28.0 |
| Blue-collar worker and craftsman | 383 | **92.0** | 88.0 | 94.7 | 34 | **8.0** | 5.3 | 12.0 |
| Clerk | 1967 | **91.0** | 88.9 | 92.8 | 172 | **9.0** | 7.2 | 11.1 |
| Intermediate worker | 1123 | **93.5** | 90.9 | 95.3 | 74 | **6.5** | 4.7 | 9.1 |
| Executive | 981 | **95.6** | 92.7 | 97.4 | 32 | **4.4** | 2.6 | 7.3 |
|  |  |  |  |  |  |  |  |  |
| **Household income (in euros)** |  |  |  |  |  |  |  |  |
| <2100 | 1420 | **88.5** | 85.7 | 90.8 | 167 | **11.5** | 9.2 | 14.3 |
| >2100 and ≤2800 | 804 | **93.2** | 90.7 | 95.0 | 60 | **6.8** | 5.0 | 9.3 |
| >2800 and ≤4200 | 1376 | **95.0** | 93.1 | 96.4 | 66 | **5.0** | 3.6 | 6.9 |
| >4200 | 927 | **95.8** | 92.6 | 97.7 | 29 | **4.2** | 2.3 | 7.4 |
|  |  |  |  |  |  |  |  |  |
| **Marital status** |  |  |  |  |  |  |  |  |
| Single | 906 | **93.7** | 90.4 | 95.9 | 52 | **6.3** | 4.1 | 9.6 |
| Married or living as a couple | 2975 | **94.3** | 92.9 | 95.4 | 163 | **5.7** | 4.6 | 7.1 |
| Separated, divorced or widowed | 646 | **84.2** | 79.8 | 87.8 | 107 | **15.8** | 12.2 | 20.2 |
|  |  |  |  |  |  |  |  |  |
| **Alcohol use disorder risk^5^** |  |  |  |  |  |  |  |  |
| Mild | 3998 | **92.6** | 91.3 | 93.8 | 282 | **7.4** | 6.2 | 8.7 |
| At-risk | 529 | **91.5** | 87.3 | 94.4 | 40 | **8.5** | 5.6 | 12.7 |
|  |  |  |  |  |  |  |  |  |
| **Depressive state^6^** |  |  |  |  |  |  |  |  |
| No | 3350 | **95.2** | 93.9 | 96.3 | 146 | **4.8** | 3.7 | 6.1 |
| Yes | 1177 | **86.0** | 83.0 | 88.6 | 176 | **14.0** | 11.4 | 17.0 |
| ^1^N: Unweighted headcount; ^2^Weighted prevalence; ^3^Confidence Interval at 95% of the weighted prevalence; ^4^Based on the 2011 International Standard Classification of Education; ^5^At-risk alcohol use disorder was defined as a total score >7 at the Alcohol Use Disorder Identification Test; ^6^Depressive state was defined as a total score >18 at the Center for Epidemiological Studies Depression Scale. Results were computed from weighted analyses of 4849 women included in 2015 in the CONSTANCES cohort. | | | | | | | | |
